# Influence of Different Frequency Power Generators on Plasma Temperatures in the Ammonia Decomposition Process: OES-Based Temperature Profiling in a Gliding Arc

**DOI:** 10.1021/acsomega.6c04814

**Published:** 2026-09-22

**Authors:** Michalina Perron, Tim Gehring, Mateusz Wiosna, Wojciech Gajewski, Michał Młotek

**Affiliations:** † Faculty of Chemistry, 49566 Warsaw University of Technology, Noakowskiego 3, Warszawa 00-664, Poland; ‡ TRUMPF Huettinger Sp. z o.o., Marecka 47, Zielonka 05-220, Poland; § TRUMPF Hüttinger GmbH + Co. KG, Bötzinger Straße 80, Freiburg 79111, Germany

## Abstract

A gliding arc plasma reactor operated with highly concentrated ammonia (90 vol % NH_3_ in N_2_) was investigated using spatially resolved optical emission spectroscopy, electrical diagnostics, and gas chromatography. Fitting the N_2_ second positive system (310–360 nm) yielded rotational and vibrational temperature profiles along the arc for 50 Hz and Bipolar HV high-frequency (25–35 kHz) excitation. Results indicate local thermal equilibrium near ignition and a transition to nonequilibrium along the arc, which is a typical characteristic of a gliding arc. Bipolar HV kHz excitation produced higher vibrational temperatures, enhanced N_2_^+^ emission downstream, and substantially increased reaction rates localized in the discharge channel. It demonstrates improved energy concentration and higher ammonia conversion compared to 50 Hz, and promising potential for energy-efficient hydrogen recovery from ammonia.

## Graphical abstract

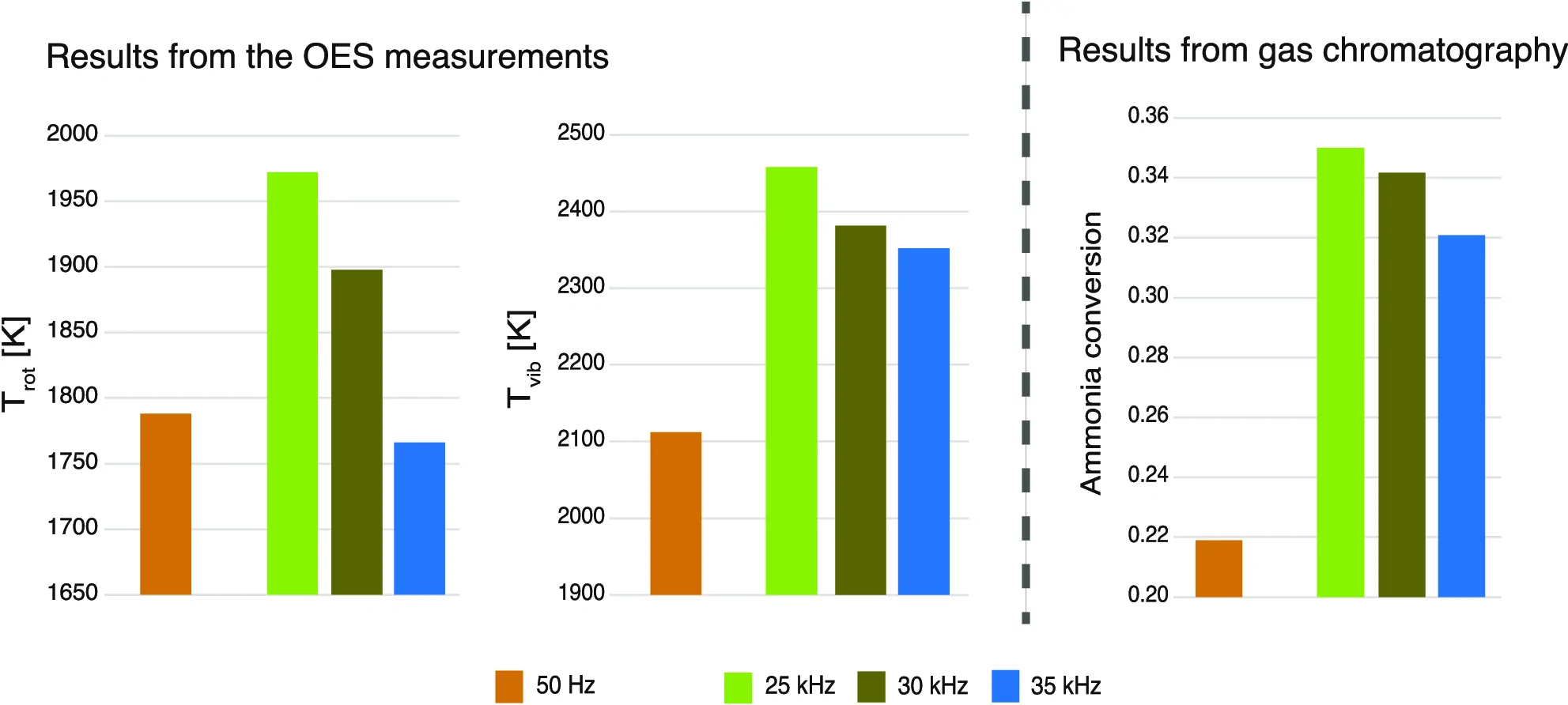

## Introduction

Ammonia is widely recognized as a promising hydrogen carrier medium due to its high H_2_ content, ease of liquefaction, and availability of infrastructure for storage and transport. To achieve carbon-neutral technologies, the development of an efficient hydrogen cycle is essential. Hydrogen produced through water electrolysis with the use of renewable energy must be stored and transported in a stable form. Developing effective methods for hydrogen recovery from its storage is fundamental to the successful integration of the H_2_ technologies into larger-scale systems.

The study of ammonia decomposition is carried out by multiple research entities applying thermal, plasma, or plasma-catalytic methods.^1,2^ The nonthermal plasma decomposition of ammonia is attracting growing interest due to its potential to be used in a hydrogen recovery system. Among the available plasma configurations, the gliding arc offers a unique combination of delivering high local energy while maintaining a low gas temperature inside the reactor, which can be advantageous for driving chemical reactions. A gliding discharge is created when a gas stream passes through a space between the electrodes. The discharge begins at the smallest gap between them and travels with the gas stream.^3^ Despite its practical potential, the behavior of ammonia in gliding arc discharge has not been thoroughly described and analyzed. Key uncertainties concern the influence of power source, frequency, and discharge stability during the decomposition of ammonia. To address this issue, a comprehensive experimental investigation was conducted, integrating optical emission spectroscopy (OES), current–voltage diagnostics, and gas chromatography.

Among the applied diagnostic techniques, the OES plays a key role in characterizing plasma-induced chemical processes. OES is a well-established, nonintrusive diagnostic technique that enables the determination of local plasma parameters such as rotational and vibrational temperatures as well as excited species composition and distributions derived from molecular band emission.^4–13^ In many studies on atmospheric-pressure plasmas, the second positive system (SPS) of N_2_ and the first negative system (FNS) of N_2_^+^ are analyzed by fitting measured spectra with simulated band systems to extract rotational and vibrational temperatures.^5–7^ For instance, Zhang et al.^6^ and Deng et al.^7^ use the N_2_ SPS to determine temperatures in highly constricted DC plasma jets by matching experimental spectra with calculated transitions. Building on this approach, OES provides spatially and temporally resolved access to the thermodynamic state of the discharge without perturbing the plasma itself. In the present work, this methodology is applied to a gliding arc plasma used for the decomposition of ammonia. An emission spectrum in the range of 310–360 nm is recorded, and the overlapping bands of the N_2_ SPS (C^3^Π_u_ → B^3^Π_g_) and the NH radical (A^3^Π → X^3^Σ^–^) are identified and fitted using the open-source MassiveOES software,^8^ based on validated spectroscopic data for N_2_ ^9–11^ and NH.^12–14^ This method enables the extraction of rotational and vibrational temperature profiles along the arc path, which are subsequently correlated with electrical diagnostics and gas chromatography data on ammonia conversion to validate how discharge dynamics and vibrational excitation influence the efficiency of ammonia decomposition in the gliding arc reactor.

## Experimental Section

Two power supplies were examined at the Warsaw University of Technology (WUT) (Warsaw, Poland): a thyristor power supply of 50 Hz AC with 4–6 kV obtained using a Fart Resinblock 2000 high-voltage transformer and TruPlasma Bipolar 4020 G2.1 HV power supply (hereinafter referred to as Bipolar HV), delivered by TRUMPF Huettinger sp. z o. o., Zielonka, Poland. The discharge power of the WUT thyristor power supply was measured with an energy meter, Orno OR-WE-512. Electrical measurements were conducted using the Tektronix DPO3034 oscilloscope, equipped with a voltage probe (Tektronix P6015A) and a current probe (Tektronix TCP0030), connected to the electrode. Gas samples for chromatographic analysis were taken downstream of the reactor after the gas was cooled. The gas was analyzed by chromatography using a Thermo Scientific Trace 1600 with a TCD detector and an Rtx-Volatile Amine column for ammonia measurements, and a Chrompack CP 9002 chromatograph with a TCD detector for hydrogen measurements. The gas temperature after the plasma zone was measured with a type K thermocouple (Figure 1).

**Figure 1:**
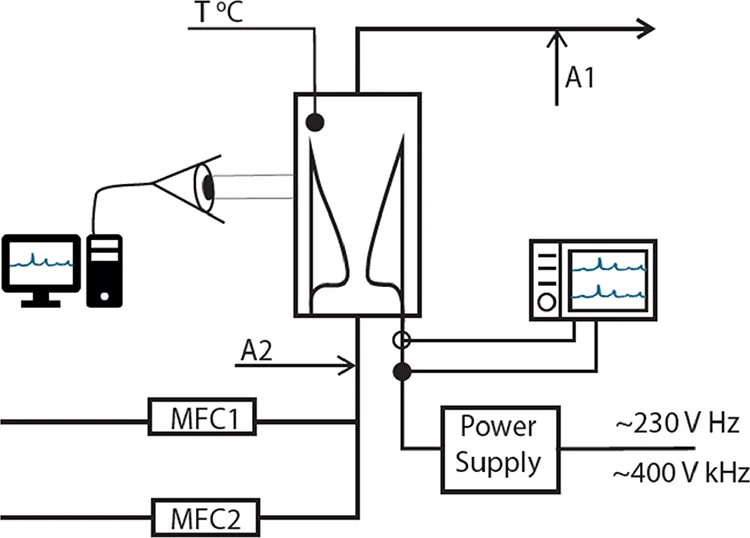
Apparatus scheme; A1, A2—gas sampling points, T—thermocouple type K, and MFC1, MFC2—mass flow rate controllers.

Nitrogen (99.999%) and ammonia (99.85%) gases were supplied by Multax. Gas streams were regulated by Bronkhorst mass flow controllers. The gas streams were mixed before entering the reactor, and the total gas flow rate was set to 180 Nl/h.

The tests were conducted at a high ammonia concentration (90% NH_3_ + 10% N_2_ by volume). The reactor was made of a quartz tube, and its volume was 0.26 dm^3^. It consists of two knife-shaped electrodes facing each other, positioned vertically and tapering toward the top. The length of the electrodes was 10 cm. The discharge was initiated where the distance between the electrodes was the narrowest, approximately 3 mm. The ammonia conversion and energy consumption were calculated using the equations below.

$$a\mathrm{mmonia}\;\mathrm{conversion}:\;x_{{NH}_{3}}=\frac{W_{0}\left[{NH}_{3}\right]-W\left[{NH}_{3}\right]}{W_{0}\left[{NH}_{3}\right]}$$

$$a\mathrm{mmonia}\;\mathrm{conversion}\;\mathrm{yield}:\;\eta_{NH_{3}}=W_{0}\left[NH_{3}\right]\cdot x_{NH_{3}}$$

$$e\mathrm{nergy}\;\mathrm{consumption}:\;E=\frac{P\cdot 3.6}{n}$$

where *W*_0_[NH_3_] ammonia inlet flow rate (mol/h), *W*[NH_3_] ammonia outlet flow rate (mol/h), *P*-discharge power (*W*).

### OES Measurements

The OES measurements were conducted using a spectrometer, AvaSpec-ULS4096CL-EVO, from Avantes B.V., equipped with an optical fiber, FC-UVIR400–2, from Avantes B.V., in a wavelength range of 300–400 nm. The spectrometer resolution is 0.5 nm (fwhm). The fiber ending was mounted on a vertical movable fixture and pointed in the plasma direction. To measure only at a defined point in the plasma area, the field of view of the fiber was limited using an aperture. The measurement setup is shown in Figure 2. Spectra were taken at seven different points inside the plasma zone, along the vertical axis in Figure 3. The discharge power was set to 300 W at frequencies of 50 Hz for the thyristor power supply and 25, 30, and 35 kHz for the Bipolar HV generator, respectively. The exact position of the measurement points can be found in Figure 3.

**Figure 2:**
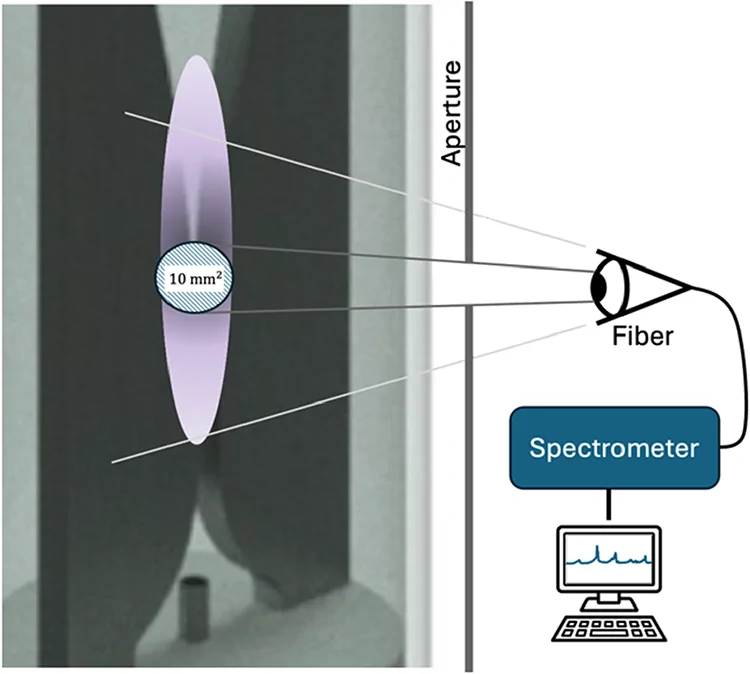
Sketch of the OES measurement setup.

**Figure 3:**
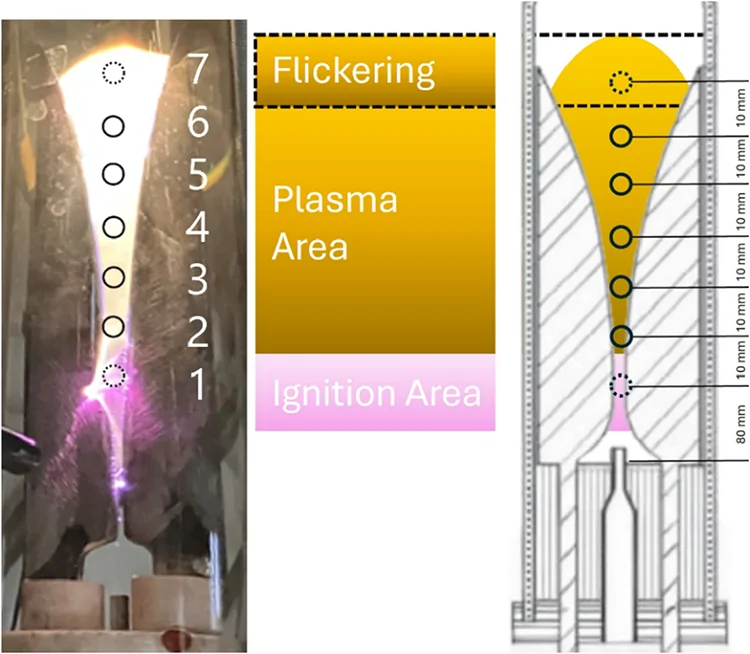
Measurement points and description of the plasma area.

The integration time of the spectrometer was set to one second and averaged over 100 measurements to ensure an averaged characteristic of the plasma at the designated point, rather than measuring the behavior of a single arc. The result of the measurement is a wavelength-resolved spectrum that represents the averaged plasma emission, averaged over the measured area, the integration time, and the number of measurements. To ensure a stabilized plasma behavior and reactor temperature. The temperature measured at the outlet of the reactor was an indicator of the achieved thermal stationary state. After that, the measurement was performed at each measurement point shown in Figure 3.

The instrumental slit (instrument response) function was estimated from the measured H-β emission by fitting a Voigt profile to the recorded spectrum. The Voigt model was used to separate instrumental (assumed Gaussian) broadening from physical line broadening (assumed Lorentzian).^4^ The fit yielded a Gaussian standard deviation σ ≈ 0.18 nm (Gaussian HWHM ≈ 0.21 nm) and a Lorentzian HWHM γ ≈ 0.1 nm. More information concerning the instrumental slit is given in the Supporting Information data.

## Results

The OES spectra were used to identify the molecular spectrum, as presented in Figure 4. Here, the spectra of the SPS and the FNS of molecular nitrogen, as well as radiation from the NH-radical transition, were found. For the determination of the rotational and vibrational temperature, the SPS (C^3^Π_u_ → B^3^Π_g_) of molecular nitrogen and the NH-radical transition (A^3^Π → X^3^Σ^–^) in the wavelength range of 310–360 nm were analyzed. The SPS was used, taking into account the overlap of the NH transition.^5–7^

**Figure 4:**
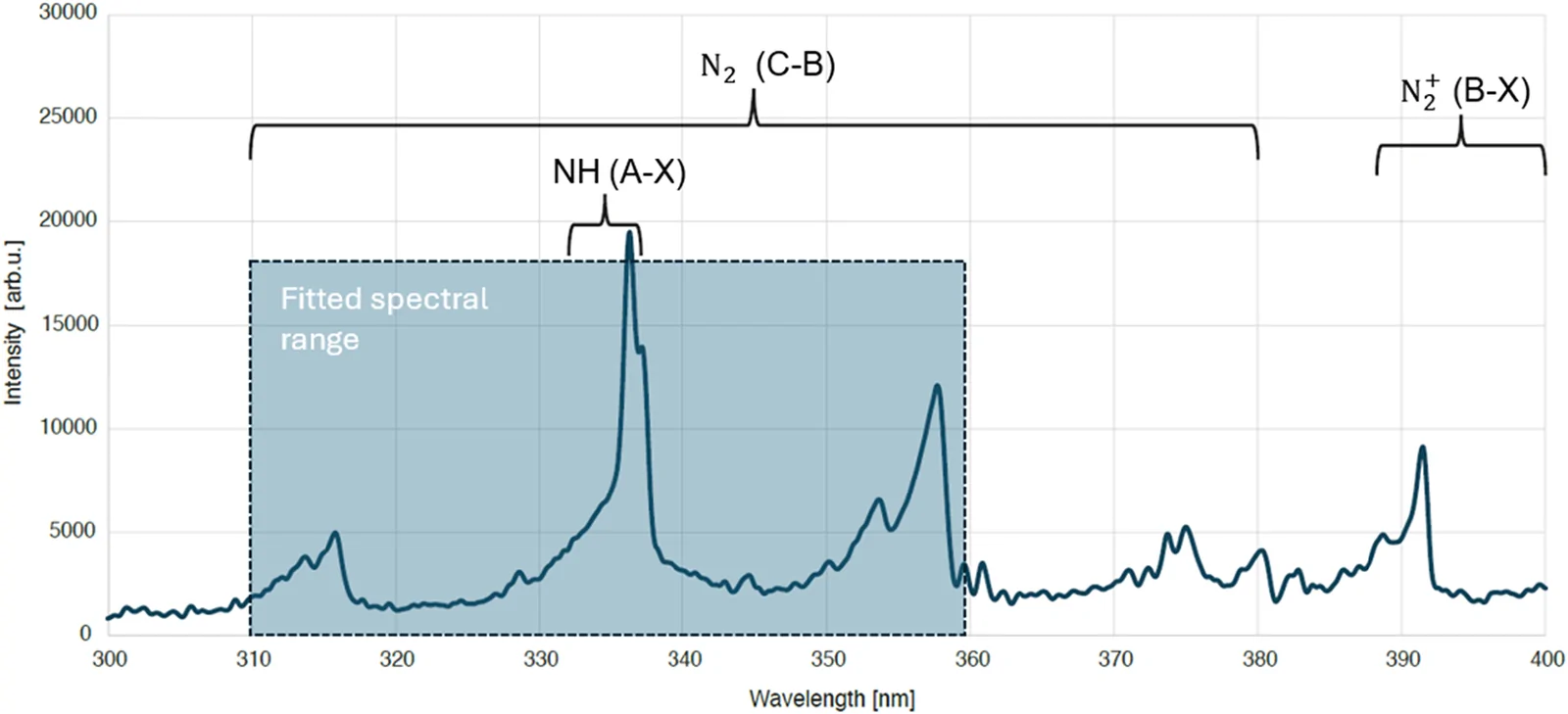
Measured spectra with indicated molecular band ranges and marked fitted wavelength range.

For the determination of temperatures, the spectra were fitted using the open-source software MassiveOES.^8^ The fits were carried out with the software spectral data for the N_2_ ^9–11^ and NH^12–14^ transitions. A more detailed description of the software and the theoretical background used for the spectral fit and temperature determination is given in the author’s description of the software.^8,15,16^ An example fit, performed at the midpoint of the plasma (position 4) with a 300 W input power and a frequency of 30 kHz, is shown in Figure 5. The rotational and vibrational temperatures determined using this method can now be compared at the assigned points when excited by the different frequencies or input powers.

**Figure 5:**
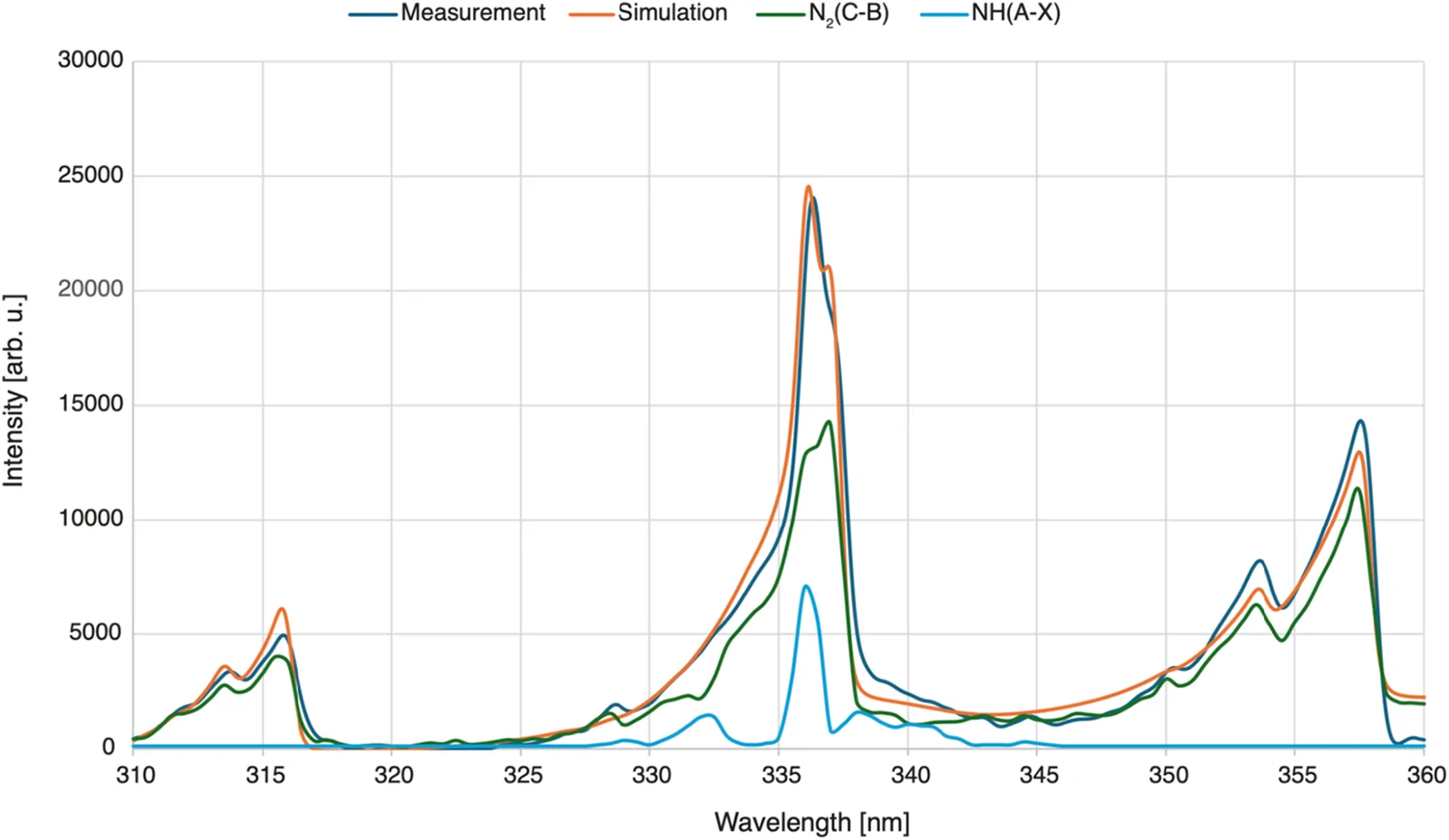
Example of a spectral fit in the wavelength range of 310–360 nm with an input power of 300 W and a frequency of 30 kHz at measured point 4.

### Frequency Dependence of Temperatures

Figure 6 depicts the temperatures obtained from the numerical fitting method at different positions along the plasma reactor. As indicated in Figure 3, point 1 is located in the area of the plasma ignition. Since the ignition position for each arc changes randomly within a certain distance range, it was not possible to take a reliable measurement at this position. This is visible from the noticeable deviation in the determined temperatures for this point. Therefore, this measuring point and the resulting temperature were excluded from the following discussion. A similar behavior is also observed at measuring point 7. Since the arc can extinguish at different points at the end, as indicated by flickering in Figure 3, a continuous measurement cannot be assumed in this case, due to fluctuations in the intensity. Therefore, this point has also been excluded from further analysis. The points 2–6 showed a continuous plasma behavior and are used to analyze the temperature distribution. It should be noted that the error bars shown in Figure 6 are the result of the deviation of the fit from the measurement and are an output of the MassiveOES software. Furthermore, given the spectral resolution available in the presented study, a precise determination of the vibrational temperature is not possible. Therefore, the conclusions do not rely on their exact values. These estimates have been included, as the obtained values are consistent with those reported in the literature.^2^

**Figure 6:**
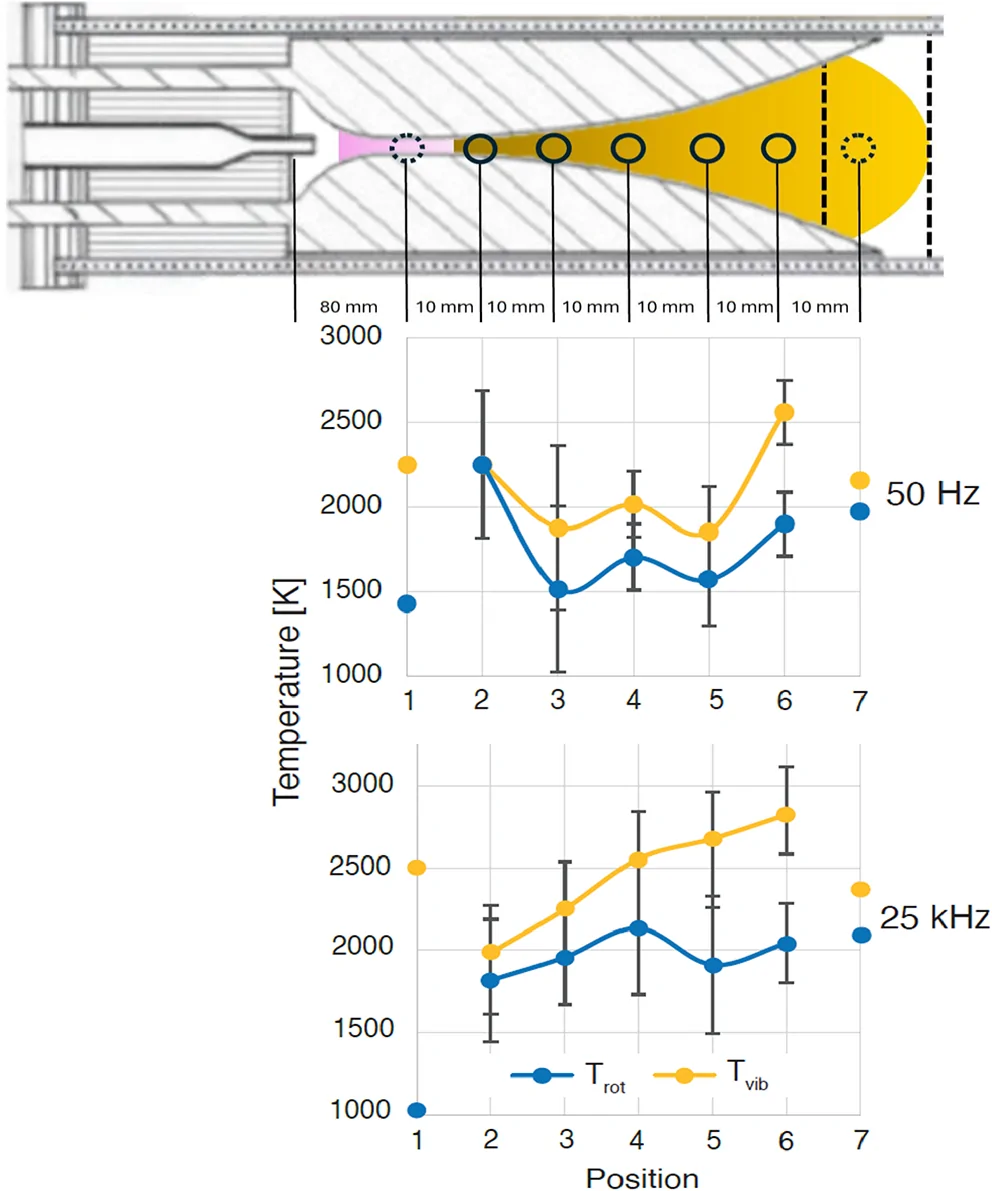
*T*_rot_ = rotational and *T*_vib_ = vibrational temperatures related to the measurement position for the 50 Hz and 25 kHz Bipolar excitation with an input power of 300 W.

The determined temperature range for the rotational temperature of *T*_rot_ ≈ 1500–2250 K for 50 Hz, 1000–2350 K for the Bipolar excitation at 25 kHz. The vibrational temperature range of *T*_vib_ ≈ 1850–2550 K and 1950–2800 K for thyristor and Bipolar, respectively, is comparable to similar experiments with gliding discharge plasma, as reported in the literature.^2^

As Figure 6 indicates, the measured temperature profiles are initially close to each other at position 2. This indicates that local thermal equilibrium can be assumed in this case. This behavior was expected and is also described in the literature.^3,17^ As the arc path continues, the difference between the vibrational and rotational temperatures increases, as can be seen especially at positions 5–6. The arc enters a region that tends toward nonequilibrium. This behavior is also known and is typical for a gliding arc discharge.^3,17^ This phenomenon was observed at all frequencies.

To verify a correlation between the temperatures and measurements of ammonia decomposition, as determined by gas chromatography, the average values of the vibrational and rotational temperatures over the arc pass at 300 W input power were calculated. This was done for all used frequencies. This approach was chosen because the gas flowing through the reactor passes through all temperature regions within the reactor. The average temperatures for the different frequencies are given in Figure 7. In addition, Table 1 summarizes the data obtained at a power of 300 W for various operating frequencies, together with the corresponding conversion levels and gas temperatures.

**Figure 7:**
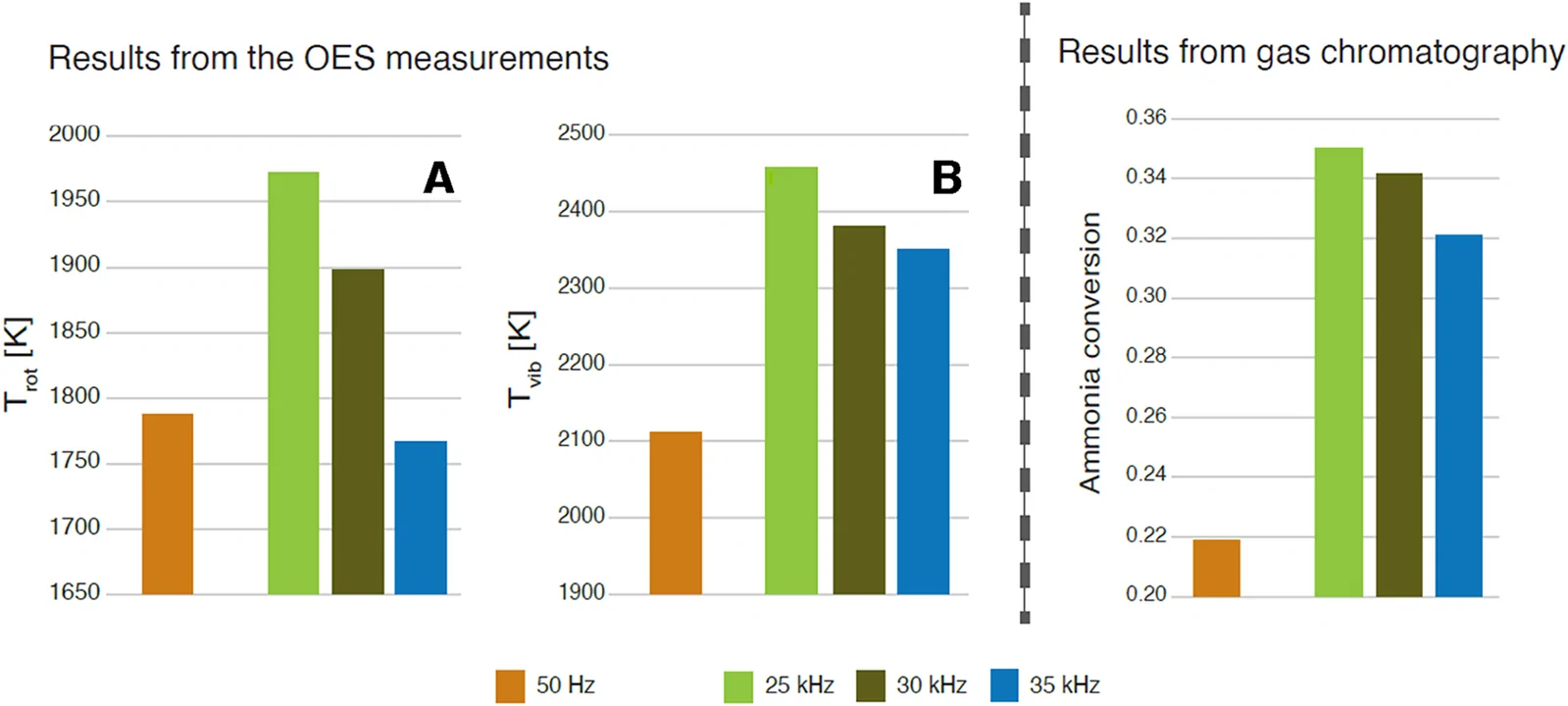
Averaged temperatures of the different excitation frequencies in comparison to the ammonia conversion. (A) Rotational temperature *T*_rot_ and (B) vibrational temperature *T*_vib_.

**Table 1:** Overview of Temperatures and Ammonia Conversion for Different Frequencies at 300 W

freq [kHz]	NH_3_ conversion	*T*_rot_ [K]	*T*_vib_ [K]	*T*_t_ [K]	*E* [kJ/molNH_3_]
0.05	0.22	1788	2112	628	679
25	0.35	1972	2457	713	427
30	0.34	1897	2318	713	439
35	0.3	1766	2352	708	498

Analyzing the rotational temperature, it can be observed that it initially rises for bipolar excitation compared to conventional excitation with a 50 Hz sinusoidal power. When the frequency is further increased, the temperature drops again. This leads to the rotational temperature at 35 kHz being lower than at 50 Hz (Figure 7A). The increase in rotational temperature when switching from 50 Hz sinusoidal excitation to 25 kHz high-voltage operation can be attributed to the different waveform characteristics.^18^ The Bipolar HV pulsed waveform delivers energy in short, high-voltage pulses with steep voltage transitions, which may promote more efficient energy transfer to the plasma and sustain repeated discharge reignition. Similar observations were reported by Mallick et al.,^19^ who demonstrated that sub-RF pulse-modulated excitation with a fast rise time concentrates a significant fraction of the deposited energy within the first oscillation cycles of each pulse, enhancing local plasma activity.

A further increase in frequency to 35 kHz resulted in a decrease in rotational temperature. This behavior may be related to the reduction in energy available per pulse with increasing repetition frequency. As discussed by Mahreen et al.,^20^ the energy delivered during a single pulse is inversely proportional to the modulation frequency $E_{p}=\frac{P\cdot D}{f_{p}}$ , where *P* is the applied power, *D* is the duty cycle, and *f*_p_ is the modulation frequency. Consequently, at higher frequencies, the discharge receives less energy per pulse and becomes shorter. Under such conditions, lower ionization efficiency and weaker plasma development may occur, leading to reduced gas heating. In agreement with this interpretation, Mahreen et al. reported a decrease in plasma plume length, emission intensity, and electron density with increasing modulation frequency. Although the discharge configurations investigated in the literature differ from the presented Bipolar HV system, both studies indicate that increasing the frequency does not necessarily enhance plasma excitation.

Analyzing the vibrational temperatures as a function of frequency, a behavior similar to rotational temperatures is observed. However, at 35 kHz, the temperature is still far above the measurement for the 50 Hz driven process (Figure 7B). If the temperature measurements are referred to the ammonia decomposition, it can be seen that the decomposition shows a clear correlation with the vibrational temperature. This behavior is to be expected, since molecules in an excited vibrational state require less additional energy to dissociate, whereby rotational temperatures play only a minor role in dissociation.^21–23^ It should be noted that the temperature analysis refers to molecular nitrogen. While nitrogen and ammonia may exhibit different rotational and vibrational temperatures, the results obtained suggest that ammonia follows similar trends under the investigated conditions. The vibrational excitation of nitrogen may additionally influence ammonia dissociation, as previous studies have shown that nitrogen dilution can enhance NH_3_ conversion.^18,24^ Since *T*_g_ ≈ *T*_rot_, the rotational temperature can be used as a measure of the gas temperature in the plasma.^25^ To determine the effect of the temperature, an investigation of the kinetic behavior was conducted.

Based on literature data,^26^ the ammonia decomposition reaction was assumed to follow first-order kinetics. The activation energy and the frequency factor of the Arrhenius equation were calculated (eq 1). Using these parameters, the constant reaction rate in the discharge channel was determined (eq 2), assuming that the rotational temperature corresponds to the gas temperature in the discharge region. Additionally, the constant reaction rate for the entire reactor was calculated based on the temperature measured with the thermocouple *T*_t_.

1 $$k={\mathrm{Ae}}^{{-E}_{a}/\mathrm{RT}}$$

2 $$r=k\left[C_{NH_{3}}\right]$$

A comparison of the constant rate at the same discharge frequency shows that the reaction rate in the discharge channel is several times higher than in the reactor volume (Table 2). This indicates that high energy can be delivered directly to the reaction zone, enabling the activation barrier to be exceeded without a significant increase in the temperature of the entire system. It should be noted that the Arrhenius analysis was performed to estimate the potential influence of localized gas heating on the ammonia decomposition. The comparison between the temperature in the discharge channel and the reactor volume was intended to illustrate the magnitude of the thermal enhancement. However, NH_3_ decomposition cannot be attributed exclusively to thermal effects, as nonequilibrium plasma conditions also promote reaction pathways involving radicals, excited species, and electrons.

**Table 2:** Reaction Rates Calculated Based on the Temperature^a^

50 Hz			25 kHz bipolar		
temp [K]		reaction rate constant	temp [K]		reaction rate constant
*T*_t_	628	5 × 10^3^	*T*_t_	713	10 × 10^3^
*T*_g_	1788	194 × 10^3^	*T*_g_	1972	233 × 10^3^

a A discharge power of 300 W.

At a constant discharge power of 300 W, higher temperatures and consequently higher reaction rates are obtained when using the Bipolar HV power supply at 25 kHz, which demonstrates its higher energy efficiency. An increase of 100 °C in reactor temperature results in a 2-fold increase in the reaction rate constant *k*(628) to *k*(713), as shown in Table 2.

It is also important to note that the reaction rate in the reactor, relative to that in the discharge channel, decreased by a factor of 39 *k*(628) to *k*(1788) for the 50 Hz power supply, whereas for the Bipolar power supply at 25 kHz, the change was by a factor of 23 *k*(713) to *k*(1972) (Table 2). This indicates that the Bipolar HV power supply enables more effective utilization of the delivered energy, leading to the formation of higher energy active species or higher concentrations of active species. The N_2_^+^ (B–X) band can serve as an indicator that the plasma, with bipolar excitation at 25 kHz, remains more energetic than with conventional 50 Hz excitation, even when reaching the more distant measuring points. If the spectrum in the range of the N_2_^+^ (B–X) band is examined at the measuring point 6 (Figure 3), it can be seen that the plasma at 25 kHz excitation still contains a clear peak of the transition around 391 nm, which is not present with conventional 50 Hz excitation (Figure 8).

**Figure 8:**
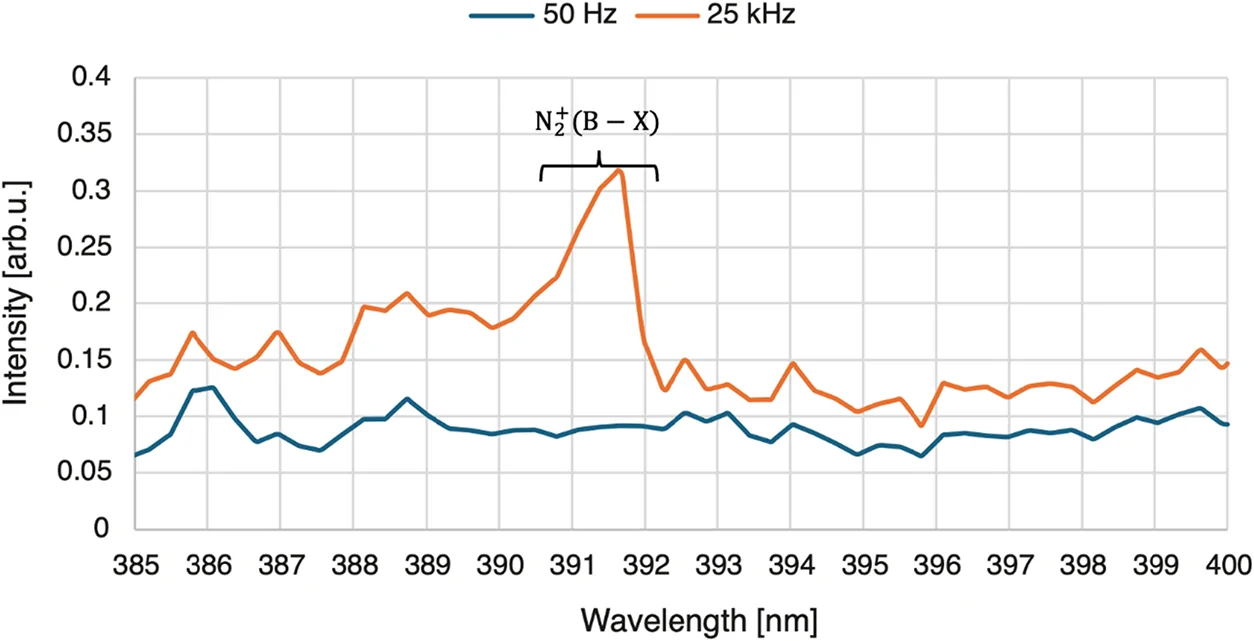
Spectral comparison of the *N*_2_^+^ (B–X) band.

## Conclusions

In this work, a gliding arc plasma reactor operated in highly concentrated ammonia (90 vol % NH_3_ in N_2_) was investigated using optical emission spectroscopy, electrical diagnostics, and gas chromatography. The combination of these methods enabled a spatially resolved characterization of the discharge and its correlation with ammonia conversion.

By fitting the N_2_ second positive system emission in the 310–360 nm range, rotational and vibrational temperature profiles along the arc path were obtained for different excitation sources, a 50 Hz thyristor and a Bipolar HV generator at 25–35 kHz.

The results show that, close to the ignition region, rotational and vibrational temperatures are similar, and local thermal equilibrium can be assumed. Along the arc trajectory, however, the increasing difference between vibrational and rotational temperatures demonstrates a transition to a nonequilibrium regime, which is characteristic of gliding arc discharge.

Averaged over the arc length, both rotational and vibrational temperatures depend on the excitation frequency and waveform. At a constant discharge power of 300 W, Bipolar HV excitation at 25 kHz yields higher vibrational temperatures and, consequently, higher ammonia conversion than conventional 50 Hz operation. Observed correlation between vibrational temperature and NH_3_ decomposition confirms that vibrationally excited molecules play a key role in the dissociation pathway, whereas the rotational gas temperature is of secondary importance under the investigated conditions.

Kinetic analysis based on first-order ammonia decomposition obtained from the temperature data shows that the reaction rate constant in the discharge channel is several times higher than the rate calculated for the temperature in the overall reactor volume. This highlights that the gliding arc effectively concentrates energy in a narrow reaction zone, overcoming the activation barrier without a strong increase in the overall reactor temperature.

Moreover, the reduction of the reaction rate from the discharge channel to the reactor volume is less pronounced for the Bipolar HV generator than for 50 Hz, indicating a more efficient utilization of the deposited electrical energy and a more persistent population of energetic species. This interpretation is supported by the presence of a distinct N_2_^+^(B–X) emission peak around 391 nm at downstream positions for 25 kHz excitation, which is absent at 50 Hz, evidencing a more energetic plasma further along the arc.

Overall, the study demonstrates that both the frequency and the waveform of the power supply have a decisive impact on the nonequilibrium character of the gliding arc and the resulting ammonia decomposition performance. Bipolar HV excitation at kHz frequencies enhances vibrational excitation and reaction rates at comparable power input, making it a promising approach for energy-efficient hydrogen recovery from ammonia in hydrogen carrier systems.

## Supplementary Material



## Notes

Approximation of the Slit Function Sensitivity Analysis of the Slit Function (PDF)
