# Supplementary material for: Influence of Different Frequency Power Generators on Plasma Temperatures in the Ammonia Decomposition Process: OES-Based Temperature Profiling in a Gliding Arc
Source: ACS Omega. 2026 Sep 22;11(39):59147–53. doi: 10.1021/acsomega.6c04814 (PMC13648063; doi:10.1021/acsomega.6c04814)
Supplement: Supplementary file 1 [file ao6c04814_si_001.pdf]

Supporting Information

Influence of Different Frequency Power Generators on Plasma Temperatures in Ammonia Decomposition Process, OES-Based Temperature Profiling in a Gliding Arc

Michalina Perron[1,2*], Tim Gehring[3], Mateusz Wiosna[2], Wojciech Gajewski[2], Michał Młotek[1]
1 Faculty of Chemistry, Warsaw University of Technology, Noakowskiego 3, 00-664 Warszawa, Poland
2 TRUMPF Huettinger Sp. z o.o., Marecka 47, 05-220 Zielonka, Poland
3 TRUMPF Hüttinger GmbH + Co. KG, Bötzinger Straße 80, 79111 Freiburg, Germany
*michalina.perron.dokt@pw.edu.pl

This Supporting Information includes:
Approximation of the Slit Function
Sensitivity Analysis of the Slit Function

<u>Approximation of the Slit Function</u>

The observed shape of a spectral line results from the superposition of several line-broadening processes. At the atomic or molecular level, natural line width (Lorentzian profile, due to the finite lifetime of excited states) and Doppler width (Gaussian profile, caused by thermal and macroscopic motions of the emitting atoms) occur [1,2]. In addition, the measured profile is distorted by the instrument: a slit, optical aberrations, end effects of the dispersive unit, and detector pixelation lead to an instrumental line shape, the slit function or instrumental profile (IP). The line $L_{obs}(\lambda)$ actually measured at the detector is approximately the convolution of the true line profile $L_{true}(\lambda)$ with the instrumental profile IP(λ) plus noise [3]:

$$L_{obs}(\lambda) = L_{true}(\lambda) * IP(\lambda) + noise$$

The Voigt profile is the mathematical convolution of a Gaussian and a Lorentz profile and is therefore well suited for describing lines in which Doppler, pressure, collision, and natural widths are all relevant [2,3].

For measurements relevant to instrumental effects, a Voigt fit to a sharp, highly emitting line (such as H-β) is useful because adjusting the Gaussian and Lorentz components and then performing a decomposition or modeling allows conclusions to be drawn about the instrumental IP. Therefore, the instrumental slit function was estimated from the measured H-β emission by fitting a Voigt profile to the recorded spectrum. The Voigt model was used to separate instrumental (assumed Gaussian) broadening from physical line broadening (assumed Lorentzian) [4]. The fit yielded a Gaussian standard deviation $\sigma \approx 0.18$ nm (Gaussian HWHM ≈ 0.21 nm) and a Lorentzian HWHM $\gamma \approx 0.1$ nm. The measured data and the Voigt fit is shown in Figure S1.

The measurement was performed in the atmospheric gliding-arc plasma. At atmospheric pressure, hydrogen Balmer lines are susceptible to significant pressure- and Stark-broadening, self-absorption and potential line asymmetries induced by local electric fields and electron density gradients. These effects increase the physical (non-instrumental) contribution to the measured line shape and complicate the separation of instrumental and plasma broadening.

Consequently, the deconvolution of the instrumental slit function from the measured profile carries larger uncertainty than under low-pressure conditions.

The absolute temperatures derived by this method should be interpreted with caution, since inaccuracies in the estimated instrumental and physical line broadenings can introduce systematic bias in the retrieved T_{rot} and T_{vib} values. In contrast, relative comparisons and trends (e.g., relative trends or temporal variations) are more robust—provided the measurement configuration and analysis procedure remain constant—and are therefore expected to reliably reflect the observed tendencies.

However, ideally, the instrumental function should be determined using an independent calibration source. As no dedicated calibration lamp was available in the present experimental setup, the instrumental response was estimated from the measured H-β profile using a Voigt fit [4].

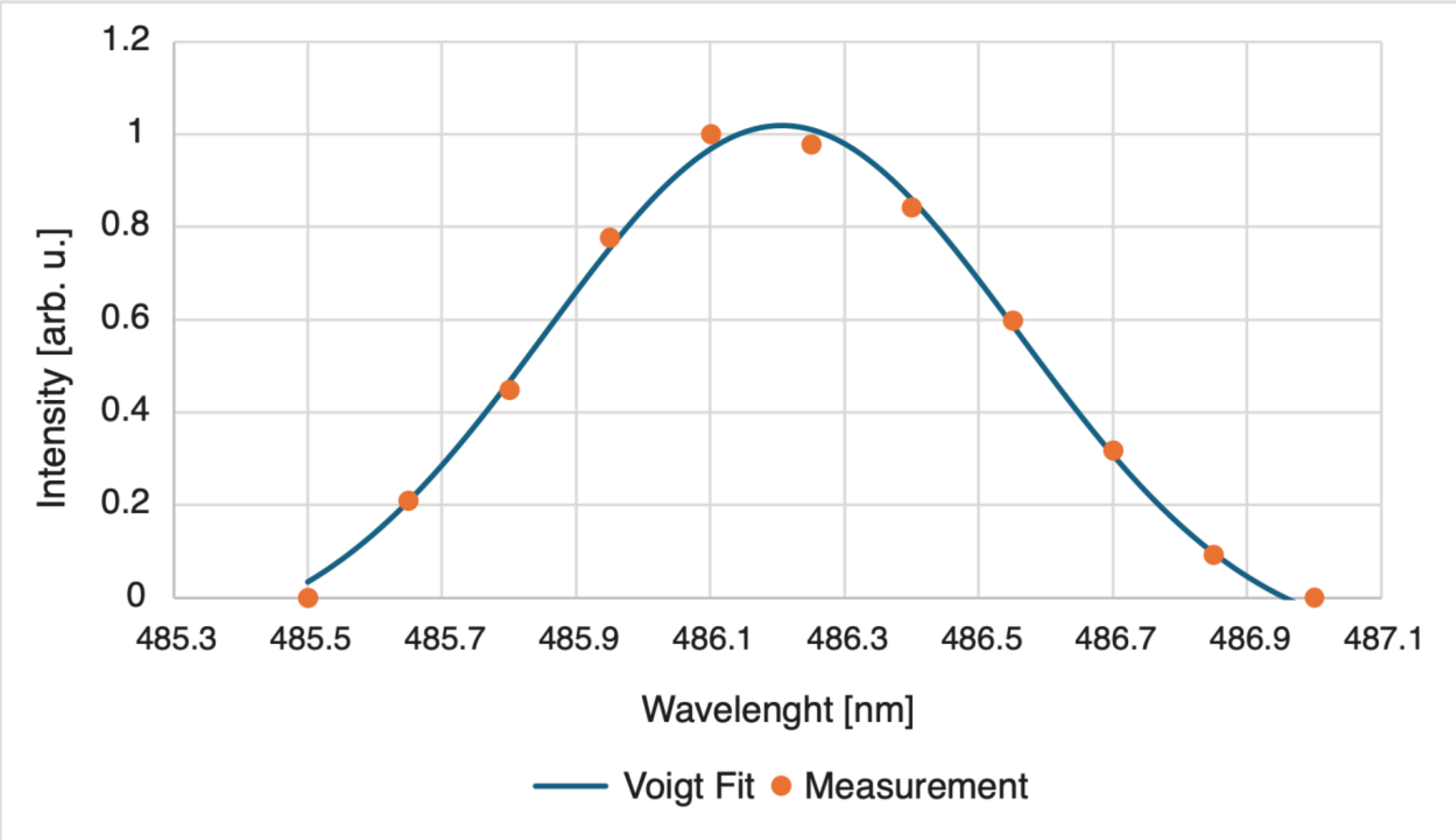

Figure S1 Normalized spectra line Hβ and corresponding Voigt-function fitting

<u>Sensitivity Analysis of the Slit Function</u>

To assess the influence of the temperature measurements against uncertainties in the determined slit function, a sensitivity analysis was performed. The slit function was varied in 5% increments from −20% to +20% around its original value, and the resulting impact on the measured rotational temperatures (T_{rot}) was analyzed across four independent measurements. The measured data and the corresponding linear fits are shown in Figure S2. The linear relationship between the slit function deviation and the temperature deviation was determined by linear regression, yielding separate fits for the negative and positive variation ranges. The resulting sensitivity formula, valid for both variation ranges, is expressed as:

$$\Delta T_{rot} = 0.8623\,|\Delta_{slit}| + 0.2857\ [\%]$$

where ΔT_{rot} denotes the deviation of the rotational temperature in percent and Δ_{slit} represents the deviation of the Gaussian HWHM from its original value in percent. As shown in Figure S2, the analysis reveals a quasi-linear, symmetric V-shaped dependence with a slope of approximately 0.86 %, indicating a near-proportional transfer of the slit function uncertainty to the derived temperature values. The standard deviation of the temperatures across the four measurements in dependence to Δ_{slit} was determined to be $\sigma_T = 2.2\%$. Since this deviation acts uniformly across all measurements, an error in the slit function introduces a systematic shift in all absolute temperature values. However, relative comparisons between measurements and temporal trends remain almost unaffected, provided that the measurement configuration and analysis procedure remain constant.

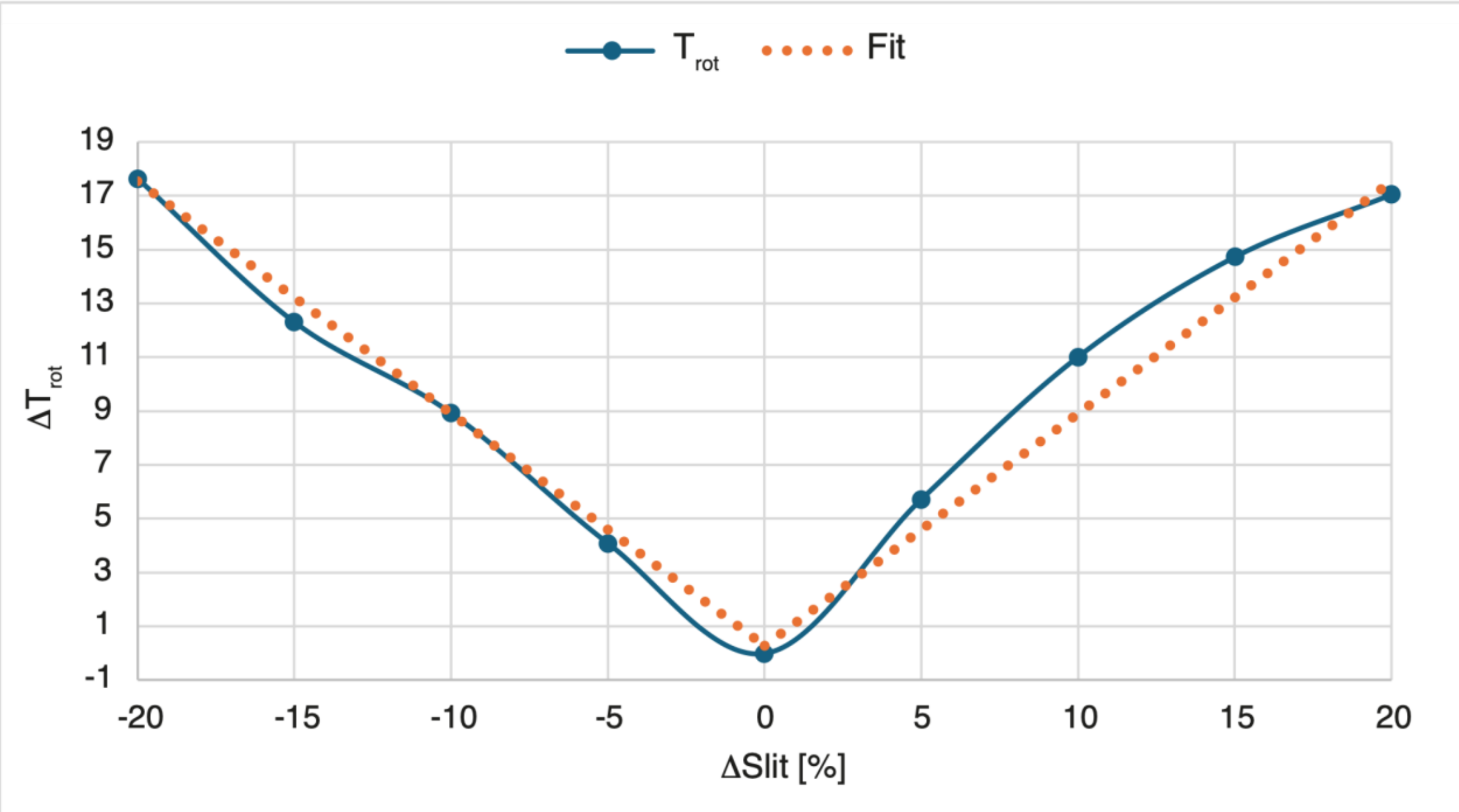

Figure S2 Dependence of rotational temperature deviation ΔT_{rot} on the Gaussian HWHM deviation (ΔSlit) from the slit function.

References

[1] Griem, H. R., *Principles of Plasma Spectroscopy*, Cambridge University Press, 1997. DOI: 10.1017/CBO9780511524578

[2] J. D. Jackson, Classical Electrodynamics (3rd ed.). Wiley, 1998. ISBN: 978-0471309321

[3] Gray, D. F., *The Observation and Analysis of Stellar Photospheres*, 3rd ed., Cambridge University Press, 2005. DOI: 10.1017/CBO9781316036570

[4] Laux, C.O.; Spence, T.G.; Kruger, C.H.; Zare, R.N. *Spectroscopic Characterization of a Pulsed Low Current High Voltage Discharge Operated at Atmospheric Pressure.* Applied Sciences 2022, 12, 6366. https://www.mdpi.com/2076-3417/12/13/6366
